# Patient preferences for using technology in communication about symptoms post hospital discharge

**DOI:** 10.1186/s12913-021-06119-7

**Published:** 2021-02-15

**Authors:** Kim E. Alexander, Theodora Ogle, Hana Hoberg, Libbie Linley, Natalie Bradford

**Affiliations:** 1 Queensland University of Technology (QUT), Centre for Healthcare Transformation, Cancer & Palliative Care Outcomes Centre, Brisbane, Australia; 2 St Vincent’s Private Hospital Northside, Brisbane, Australia

**Keywords:** Technology, Digital health, Symptoms, Communication, Health services, Patient preference, Patient discharge, Text messaging, Videoconferencing, Mobile applications

## Abstract

**Background:**

Technology is increasingly transforming the way we interact with others and undertake activities in our daily lives. The healthcare setting has, however, not yet realised the potential of technology solutions to facilitate communication between patients and healthcare providers. While the procedural and policy requirements of healthcare systems will ultimately drive such solutions, understanding the preferences and attitudes of patients is essential to ensure that technology implemented in the healthcare setting facilitates communication in safe, acceptable, and appropriate ways. Therefore, the purpose of this study was to examine patient preferences for using technology to communicate with health service providers about symptoms experienced following discharge from the hospital.

**Methods:**

Primary data were collected from patients admitted to a large metropolitan hospital in Australia during three consecutive months in 2018. Participants were asked about their daily use of technology including use of computers, email, phone, text messaging, mobile applications, social media, online discussion forums, and videoconference. They were then asked about their use of technologies in managing their health, and preferences for use when communicating about symptoms with health service providers following discharge from hospital.

**Results:**

Five hundred and twenty-five patients with a wide range of differing clinical conditions and demographics participated. Patients indicated they used a range of technologies in their everyday lives and to manage their health. Almost 60% of patients would prefer to return to hospital if they were experiencing symptoms of concern. However, if patients experienced symptoms that were not of concern, over 60% would prefer to communicate with the hospital via telephone or using technology. Admitting condition, income, and age were significantly associated with preferences for communication about symptoms following hospital discharge.

**Conclusions:**

Patients have varied preferences for communicating with their health service providers post-hospital discharge. Findings suggest that some, but not all patients, would prefer to use technology to traditional methods of communicating with the healthcare team. Health services should offer patients multiple options for communicating about their recovery to ensure individual needs are appropriately met.

**Supplementary Information:**

The online version contains supplementary material available at 10.1186/s12913-021-06119-7.

## Background

Information and communication technologies (ICT), such as telehealth, electronic patient-reported outcome measures (ePROMs), and patient portals for electronic medical records (EMR), hold great promise in enabling more efficient and effective healthcare service delivery [1]. Despite recognition that ICT enhances communication possibilities, health services have not yet realised the full potential of technologies in practice [2]. Factors identified by healthcare workers critical to adoption include: ease of technology use, integration with workflow, evidence of positive impact on patient outcomes, ability to interact with others, data security, and resourcing [3–6]. Where ICT is used in clinical practice, it has primarily been unilateral in that the health service or clinician initiates communication with patients, or uses technology in health management without patient involvement [7]. With growing demands on health services, and as patients are increasingly encouraged to engage more in self-management of their health [8], understanding patient preferences for initiating communication with health service providers warrants exploration.

Information and communication technologies are now part of almost every aspect of daily life. Alongside the proliferation of ICT across the world is the rise in their use in healthcare [9]. With the emergence of the COVID-19 pandemic, telehealth remote monitoring and consultation are now commonplace [10]. Other technologies such as ePROMs assist in systematically and comprehensively monitoring and improving symptoms resulting in reduced data entry burden for clinicians [11–13]. Patient portals for EMRs can assist with clinical history documentation, clinician prescribing, and messaging between healthcare staff and patients [7]. A review of studies about patient preferences for discharge communication found that research to date has only evaluated the provision of discharge information at the time of discharge (e.g. written or verbal summaries provided by the health service provider) [14]. Only one study has surveyed patients with hypertension in the outpatient clinic setting about use and preferences of ICTs for information and communication. Still, participants were not asked about their preferences relative to more traditional modes of communication (i.e., in-person or via a telephone call) [15]. No studies have been conducted about patient preferences for using ICTs to facilitate communication between patients and health service providers following their discharge from hospital.

The time following discharge from an admission to hospital is of great importance to recovery. Patients with different clinical conditions may experience a range of symptoms following discharge. It can be difficult for some patients to determine which symptoms are of clinical significance (i.e., whether a symptom warrants intervention by their healthcare provider) [16]. A symptom that is not of particular concern for a patient may be of significance to their healthcare. The ability for effective two-way communication about symptoms is, therefore, of great importance to the patient and the clinician. With the increasing availability of digital technologies, the possibility exists for patients to communicate about health needs electronically from the comfort of their homes, avoiding unnecessary trips to the hospital, saving both time, travel, and costs [17]. Little is known about individuals’ preference for communicating with health service providers through the use of digital technologies. The purpose of this paper, therefore, is to report on patient experience with, and preferences for, using a variety of technologies to communicate with health service providers about symptoms following discharge from the hospital. Further we seek to explore demographic factors that may be helpful in identifying patients with unique communication preferences.

## Methods

### Design and participants

Patients arriving for a planned admission to a metropolitan hospital in Queensland during three consecutive months in 2018 were surveyed in the hospital admission area about their technology use and preferences. Patients were given verbal and written information about the study and provided with a paper survey. Verbal and implied consent was obtained from all participants. Patients interested in participating did so by voluntarily and anonymously completing the survey and returning it to the research team via collection boxes. This form of informed consent was used because of the low-risk nature of the study, to reduce time pressures in participation, and to ensure participant anonymity. A waiver of written consent for this study met national regulations and was approved by the Institutional Research Ethics Committee.

### Measures

A descriptive survey was developed for this study and is provided as Additional File 1. The survey included self-reported questions about current use of technology, and preferences for the use of technology when communicating with their healthcare team post-hospital discharge about symptoms. The questions about the use of technology asked patients about their use of 11 types of digital technologies in their general day to day activities, and for managing health, as well as their future interest in using digital technologies. Participants could answer with one of three options: 1) ‘I currently use’, 2) ‘I don’t use but would be interested in using’, and 3) ‘I don’t use and don’t have any interest in using’. The survey also asked participants to rank their preference of nine options for communicating about symptoms with the healthcare team following discharge.

The survey included self-reported questions about patient demographic and clinical characteristics. These variables were asked because they are common demographic and clinical variables collected by most medical record systems and may be useful in identifying people with unique communication preferences. For most characteristics (i.e., gender, age, highest educational training, language spoken at home, employment, annual household income, living arrangements, and responsibilities for others at home), participants were asked to select a category of those presented in Table 1. For the characteristic ‘geographic area of residence’, participants were asked to provide their postal (zip) code. The code was then categorised based on the Australian Standard Geographical Classification System (i.e., ‘metropolitan’, ‘inner regional’, ‘outer regional/remote’). Participants were asked to write in free-text their condition requiring treatment and planned treatment. Responses were then categorised by two clinicians based on the hospital speciality areas (i.e., ‘orthopaedic’, ‘cardiac’, ‘oncological’, ‘gastrointestinal’ or ‘other’), and if the treatment involved ‘surgery’ or was for ‘investigation or medical care’, respectively. Members from the hospital patient advisory group provided input about the survey readability, and the survey was pilot tested with 33 admitted medical patients. Minor wording modifications were made to the survey after testing. Responses collected during the pilot testing were excluded in the final analyses.

**Table 1:** Characteristics of patients that presented to hospital for a planned admission and participated in the study

Characteristic		Respondents (*n* = 525)	%
Gender	Male	244	50.6
	Female	238	49.4
Age group (years)	18–30	28	6.7
	31–50	97	23.2
	51–65	136	32.5
	66–80	135	32.2
	81+	23	5.5
Geographic area of residence	Metropolitan	353	73.5
	Inner regional	98	20.4
	Outer regional/remote	29	6.0
Condition requiring treatment	Orthopaedic	142	27.0
	Cardiac	75	14.3
	Oncological	41	7.8
	Gastrointestinal	111	21.1
	Other	74	14.1
Planned treatment	Surgery	326	68.1
	Investigation/medical care	153	31.9
Highest Educational Training	School	229	44.5
	Vocational	154	30.0
	Tertiary	131	25.5
Language spoken at home	English	503	97.9
	Other	11	2.1
Employment	Employed	284	55.4
	Unemployed	19	3.7
	Retired	210	40.9
Annual Household Income (AUD)	< 100,000	314	67.4
	≥ 100,000	152	32.6
Living arrangements	Lives with others	456	88.9
	Lives alone	57	11.1
Responsibilities for children at home	No	393	76.3
	Yes	122	23.7
Responsibilities for elders at home	No	485	94.2
	Yes	30	5.8

### Data analysis

SPSS (Version 25) was used to perform data analyses. Descriptive statistics were primarily used in this study to describe the responses to most survey questions. To compare patient preferences for using digital technologies in relation to more traditional methods for communicating with health service providers following discharge from hospital, crude relationships between patient characteristics and communication preferences, categorised as ‘in-person’, ‘by telephone’ or ‘through digital technology ’, were established using Chi-square analyses. Multinominal logistic regression was used to identify potentially significant demographic predictors of various communication preferences (telephone compared to in-person and digital technology compared to in-person) and included significant (*p* < 0.05) covariates identified in the bivariate analyses. Age and gender were retained in the model as historical variables of interest relating to preferencing. Relative Risk Ratios (RRR), Confidence Intervals (CI), and *p*-values are presented. As the focus of the analyses was exploratory, not to test or build a predictive model, standard (simultaneous) multiple regression analysis was used over other regression models and associated model building techniques.

## Results

During the period of the survey there were 2401 unique planned admissions at the hospital. A total of 603 surveys were returned. Due to significant missing data about their current technology use and preferences 78 surveys were removed. The results for this study included responses from 525 patients with a planned admission to the hospital. Table 1 provides the characteristics of the sample. There was an approximately equal distribution of male (*n* = 244, 51%) and female (*n* = 238, 49%) patients, and the majority (*n* = 294, 70%) were aged over 50 years, and most (*n* = 503, 98%) spoke English at home. Only 26.4% of participants lived in a regional/remote area (i.e., an area that is more than a one-hour drive to the hospital). Many (*n* = 285, 54%) had completed vocational or postgraduate studies and a third (*n* = 152, 33%) had an annual household income above 100,000 AUD. The majority lived with other people (*n* = 456, 89%), but did not have responsibilities for children (*n* = 393, 76%) or elders (*n* = 485, 94%) at home. Reasons for admission were for surgery (e.g., knee replacement, breast cancer surgery) (*n* = 326, 68%) or for investigational procedures and medical care (e.g., angiogram, cystoscopy) (*n* = 153, 32%). Patients were admitted for a variety of conditions including gastrointestinal (*n* = 111, 21%), orthopaedic (*n* = 142, 14%), cardiac (*n* = 75, 14%), and oncological (*n* = 41, 8%).

Patients reported using a range of technologies as part of their general day to day activities (Table 2). The most frequently reported use was mobile phone (*n* = 495, 97%), text messaging (*n* = 454, 93%), email (*n* = 452, 93%), and the internet or websites (*n* = 451, 93%). The least frequently used was online discussion groups or forums (*n* = 152, 40%). Patients also reported high use of technology to assist in managing their health (see Table 2). For example, the most frequently reported use was mobile phone (*n* = 365, 82%), internet/websites (*n* = 320, 78%), email (*n* = 325, 93%), a laptop or desktop computer (*n* = 317, 74%), and text messaging (*n* = 275, 76%). Using a tablet or mobile phone application to assist in managing health was the most frequently reported technology to be of interest to those not currently using (*n* = 70, 20%). However, across all technologies, more patients were not interested than those who were interested in using each technology.

**Table 2:** Use of technology in general daily activities and to manage health

	Use of technology in general daily activities						Use of technology to manage health					
	Currently use		Do not use but interest in using		Do not use and no interest in using		Currently use		Do not use but interest in using		Do not use and no interest in using	
	n	%	n	%	n	%	n	%	n	%	n	%
Laptop or desktop computer	430	89.2	15	3.1	37	7.7	317	74.2	41	9.6	69	16.2
Tablet	321	75.5	46	10.8	58	13.6	204	56.4	72	19.9	86	23.8
Internet or websites	451	93.2	8	1.7	25	5.2	320	77.7	34	8.3	58	14.1
Email	452	93.0	13	2.7	21	4.3	325	77.6	38	9.1	56	13.4
Mobile phone	495	97.2	4	0.8	10	2.0	365	82.2	31	7.0	48	10.8
Home phone	321	73.0	6	1.4	113	25.7	194	54.6	25	7.0	136	38.3
Text messaging	454	93.2	10	2.1	23	4.7	275	70.5	50	12.8	65	16.7
Mobile phone or tablet applications	360	82.8	25	5.7	50	11.5	191	54.4	70	19.9	90	25.6
Online social networking services	328	73.8	9	2.0	107	24.1	126	37.7	32	9.6	176	52.7
Online discussion group or forum	152	40.2	37	9.8	189	50.0	81	25.2	53	16.5	188	58.4
Tele/video conferencing	189	47.8	57	14.4	149	37.7	67	21.1	75	14.3	175	55.2

Patient-ranked communication preferences regarding symptoms post-discharge from the hospital for a planned admission are presented in Table 3. For symptoms of little concern, telephoning the hospital was the most common first preference (*n* = 193, 37%), followed by attending the hospital in-person (*n* = 179, 34%). Approximately 30% (*n* = 160) of patients ranked a type of technology as their first preference for communication about symptoms that were of little concern. For symptoms of concern, in-person communication was the most common preference (*n* = 305, 58%), followed by communicating by telephone (*n* = 168, 32%). Communicating with any other technologies was the first preference by only 10% (*n* = 49) of respondents. In terms of the types of technology, the least common preferred option for both symptoms of low and higher concern was for online discussion forums.

**Table 3:** Communication preferences of patients about symptoms with the healthcare team post hospital discharge

	Preferences for symptoms not of concern												Preferences for concerning symptoms											
	1st			2nd			3rd			9th			1st			2nd			3rd			9th		
	Rank	n	%	Rank	n	%	Rank	n	%	Rank	n	%	Rank	n	%	Rank	n	%	Rank	n	%	Rank	n	%
Telephone	1	193	36.8	1	160	30.5	4	57	12.4	7	2	0.4	2	168	32.0	1	258	54.3	5	32	7.2	5	10	1.8
In-person	2	179	34.1	4	58	12.1	5	46	10.0	4	14	2.7	1	305	58.1	2	99	20.8	6	21	4.7	6	9	2.2
Email	3	86	16.4	2	96	20.0	2	101	21.9	9	0	0.0	3	22	4.2	3	39	8.2	1	121	27.1	9	1	0.2
Text message	4	43	8.2	3	91	18.93	1	107	23.2	6	6	1.1	4	12	2.3	4	27	5.7	2	111	24.8	7	7	1.3
Online via laptop or computer	5	13	2.5	7	22	4.6	3	58	12.6	8	1	0.2	7	2	0.4	6	10	2.1	4	42	9.4	8	4	1.0
Tele/video conference	6	7	1.3	5	27	5.6	7	39	8.5	3	40	7.6	5	6	1.1	5	34	6.5	3	101	22.6	4	29	7.0
Online via mobile phone or tablet applications	6	7	1.3	6	24	5.0	6	45	9.8	5	7	1.3	6	3	0.6	7	7	1.5	7	16	3.6	2	231	41.3
Online via social networking services	7	2	0.4	9	1	0.2	8	7	1.5	2	130	24.8	8	1	0.2	8	1	0.2	8	3	0.7	3	129	24.6
Online via discussion group or forum	7	2	0.4	8	2	0.4	9	1	0.2	1	222	42.3	6	3	0.6	9	0	0.0	9	0	0.0	1	228	43.4

Bivariate analyses (Table 4) identified significant associations between several variables. Age (*p* = .0001), condition requiring treatment (*p* = .02), admitting medical condition (*p* = .02), employment (*p* = .0001), and household income (*p* = .01) were associated with differing preferences for communicating about symptoms that were not of concern following hospital discharge. Type of condition requiring treatment was the only variable associated with preferences for communicating about symptoms that were of concern (*p* = .01).

**Table 4:** Characteristics associated with preferences for communicating about symptoms post hospital discharge

Characteristic		Preferences for symptoms not of concern								Preferences for symptoms of concern							
		In-person		Telephone		Technology^a^		χ^2^	Sig.	In-person		Telephone		Technology^a^		χ^2^	Sig.
		n	%	n	%	n	%			n	%	n	%	n	%		
Gender	Male	95	38.9	84	34.4	65	26.6	3.63	0.16	145	59.4	70	28.7	29	11.9	1.35	0.51
	Female	73	30.7	92	38.7	73	30.7			139	58.4	77	32.4	22	9.2		
Age group (years)	18–30	8	28.6	8	28.6	12	42.9	26.44	0.001	21	75.0	6	21.4	1	3.6	11.59	0.17
	31–50	27	27.8	39	40.2	31	32.0			61	62.9	26	26.8	10	10.3		
	51–65	43	31.6	39	28.7	54	39.7			87	64.0	37	27.2	12	8.8		
	66–80	60	44.4	51	37.8	24	17.8			73	54.1	48	35.6	14	10.4		
	81+	10	43.5	11	47.8	2	8.7			9	39.1	12	52.2	2	8.7		
Geographic area	Metro	128	36.3	126	35.7	99	28.0	1.11	0.89	219	62.0	104	29.5	30	8.5	7.04	0.13
	Inner Regional	30	30.6	38	38.8	30	30.6			53	54.1	30	30.6	15	15.3		
	Outer Regional/Remote	10	34.5	11	37.9	8	27.6			13	44.8	12	41.4	4	13.8		
Condition requiring treatment	Orthopaedic	43	30.3	57	40.1	42	29.6	18.66	0.02	74	52.1	45	31.7	23	16.2	19.15	0.01
	Cardiac	39	52.0	15	20.0	21	28.0			51	68.0	18	24.0	6	8.0		
	Oncological	11	26.8	18	43.9	12	29.3			19	46.3	18	43.9	4	9.8		
	Gastrointestinal	33	29.7	45	40.5	33	29.7			76	68.5	30	27.0	5	4.5		
	Other	31	41.9	28	37.8	15	20.3			47	63.5	22	29.7	5	6.8		
Planned treatment	Surgery	95	29.1	129	39.6	102	31.3	14.74	0.001	196	60.1	98	30.1	32	9.8	0.78	0.68
	Investigation/medical care	72	47.1	46	30.1	35	22.9			88	57.5	46	30.1	19	12.4		
Highest Educational Training	School	86	37.6	86	37.6	57	24.9	5.36	0.25	121	52.8	80	34.9	28	12.2	7.04	0.13
	Vocational	45	29.2	57	37.0	52	33.8			89	57.8	48	31.2	17	11.0		
	Tertiary	44	33.6	44	33.6	43	32.8			88	67.2	32	24.4	11	8.4		
Employment	Employed	85	29.9	98	34.5	101	35.6	20.38	< 0.001	176	62.0	74	26.1	34	12.0	8.16	0.09
	Unemployed	2	10.5	9	47.4	8	42.1			9	47.4	7	36.8	3	15.8		
	Retired	88	41.9	80	38.1	42	20.0			113	53.8	78	37.1	19	9.0		
Annual Household Income (AUD)	< 100,000	115	36.6	111	35.4	88	28.0	9.90	0.01	179	57.0	102	32.5	33	10.5	1.49	0.48
	≥ 100,000	34	22.4	62	40.8	56	36.8			93	61.2	41	27.0	18	11.8		
Living arrangements	Lives with others	161	35.3	161	35.3	134	29.4	1.93	0.38	271	59.4	134	29.4	51	11.2	4.96	0.08
	Lives alone	15	26.3	24	42.1	18	31.6			27	47.4	25	43.9	5	8.8		
Children at home	No	135	34.4	141	35.9	117	29.8	0.14	0.93	221	56.2	128	32.6	44	11.2	2.31	0.31
	Yes	41	33.6	46	37.7	35	28.7			78	63.9	32	26.2	12	9.8		
Elders at home	No	162	33.4	177	36.5	146	30.1	2.51	0.29	280	57.7	149	30.7	56	11.5	3.94	0.14
	Yes	14	46.7	10	33.3	6	20.0			19	63.3	11	36.7	0	0.0		

^a^The technology category consists of email, text message, online via laptop or computer, tele/video conference, online via mobile phone or tablet applications, online via social networking services, online via discussion group or forum

In the multivariable analyses, after controlling for salient covariates (i.e., those identified through bivariate analyses as well as age and gender), the admitting condition, income, and age remained significantly associated with communication preferences about symptoms following hospital discharge (Table 5). Type of treatment received and employment were not included in the multivariable model because of the potential for multicollinearity with condition receiving treatment and annual household income, respectively. For symptoms not of concern, those aged 66–80 years had a decreased preference for using technology to communicate than in-person compared to those aged between 51 and 65 years (RRR 0.40; CI 0.19, 0.83). Also, having either a cardiac or ‘other’ condition compared to having an orthopaedic condition was associated with decreased preference for in-person communication than a telephone call (RRR 0.19; CI 0.08–0.45, RRR 0.44; CI 0.20, 0.98, respectively). Having a household income of more than 100,000 AUD per year was associated with increased preference for telephone and technology than in-person modes of communication about symptoms of low concern (RRR 2.43; CI 1.25, 4.74, RRR 2.09; CI 1.08, 4.07, respectively). In comparison to those aged between 51 and 65 years, those aged over 80 years had a greater preference for telephone than in-person to communicate about symptoms of concern (RRR 2.72; CI 1.01, 9.34). Lastly, patients with gastrointestinal conditions had a decreased preference for in-person communication than using technology to communicate about symptoms of concern compared to patients with orthopaedic conditions (RRR 0.28; CI 0.09, 0.91).

**Table 5:** Multivariable analysis of characteristics associated with preferences for communicating by telephone or by using technology compared to in-person communication about symptoms post hospital discharge

Characteristic		Telephone compared to In-Person Communication			Technology^a^ compared to In-Person Communication		
		RRR	CI 95%	Sig.	RRR	CI 95%	Sig.
Preferences for symptoms not of concern (*n* = 341)							
Gender	Male	REF			REF		
	Female	1.24	0.71, 2.17	0.45	1.09	0.61, 1.94	0.77
Age	18–30	0.47	0.12, 1.81	0.27	1.15	0.38, 3.47	0.81
	31–50	0.88	0.41, 1.88	0.73	0.65	0.30, 1.41	0.28
	51–65	REF			REF		
	66–80	0.98	0.49, 1.97	0.96	0.40	0.19, 0.83	0.01
	81+	2.32	0.70, 7.74	0.17	0.33	0.06, 1.83	0.21
Condition requiring treatment	Orthopaedic	REF			REF		
	Cardiac	0.19	0.08, 0.45	< 0.0001	0.50	0.22, 1.13	0.10
	Oncological	0.90	0.32, 2.50	0.84	1.14	0.38, 3.45	0.81
	Gastrointestinal	0.67	0.32, 1.39	0.28	0.70	0.31, 1.45	0.31
	Other	0.44	0.20, 0.98	0.05	0.45	0.18, 1.08	0.07
Annual Household Income (AUD)	< 100,000	REF			REF		
	≥ 100,000	2.43	1.25, 4.74	0.01	2.09	1.08, 4.07	0.03
		OR	CI 95%	Sig.	OR	CI 95%	Sig.
Preferences for symptoms of concern (*n* = 382)							
Gender	Male	REF			REF		
	Female	1.23	0.77, 1.97	0.39	0.97	0.45, 2.10	0.94
Age	18–30	0.36	0.10, 1.30	0.12	0.55	0.06, 4.70	0.58
	31–50	0.95	0.50, 1.78	0.87	1.44	0.52, 3.95	0.48
	51–65	REF			REF		
	66–80	1.45	0.82, 2.57	0.20	1.28	0.50, 3.33	0.62
	81+	2.72	1.01, 7.34	0.05	1.70	0.31, 9.31	0.54
Condition	Orthopaedics	REF			REF		
	Cardiology	0.53	0.26, 1.09	0.08	0.62	0.22, 1.78	0.37
	Oncology	1.19	0.53, 2.65	0.68	0.95	0.27, 3.33	0.94
	GI	0.69	0.37, 1.28	0.24	0.28	0.09, 0.91	0.04
	Other	0.71	0.36, 1.42	0.34	0.43	0.13, 1.40	0.16

*RRR* Relative Risk Ratio, *REF* Referent group (largest category), ^a^ The technology category consists of email, text message, online via laptop or computer, tele/video conference, online via mobile phone or tablet applications, online via social networking services, online via discussion group or forum

## Discussion

Consistent with reports of increasingly widespread use of communication technology in society, patients in this study reported using a wide variety of communication technologies in their daily activities [18]. At least half of the patients in this study reported using some technologies such as computers, the internet, and the telephone (including text messaging) to manage their health. This finding is consistent with reported trends of individuals’ rapidly increasing uptake of technology, such as the internet, to manage their health [19]. However, only a small proportion of patients were interested in using new technologies that they were not currently using in general daily activities in managing their health. The reluctance to utilise more technology-enabled approaches in the context of healthcare may be indicative of a lack of experience with the technology for health management [20–22], and concerns about privacy [22, 23]. It may also be reflective of an older demographic of patients who may have misconceptions about the difficulty of using technology, or issues with trust [24].

Our findings show differences in patient preferences for communicating with the healthcare team post-hospital discharge following a planned admission. Nearly 60% of patients preferred to return to hospital to communicate about symptoms that were of concern to them, but at least 30% preferred to communicate via telephone. If patients experienced symptoms that did not cause them concern, two-thirds preferred to use either telephone or other technology to communicate with the health service. These results may suggest that many patients would prefer not to return to the hospital for follow-up unless they were experiencing symptoms of concern. Our findings should not only prompt renewed interest in the role of follow-up telephone calls for patients discharged from the hospital [25] but the role of virtual follow-up visits with health services [26].

In the current study, having a cardiac condition was associated with preferencing in-person communication over a telephone call. Having a gastrointestinal condition was also associated with a decreased preference for using technology to communicate when concerned about symptoms. Perhaps the experience of specific conditions is associated with higher treatment-seeking behaviour and the need for more urgent attention. Tran and colleagues [27] found that patients with concerns about cardiac symptoms were more likely to self-present to the emergency department despite receiving telephone review and health helpline advice that their symptoms were of ‘low urgency’ and ‘require self-care’. All orthopaedic patients received a planned surgical intervention. While all the other conditions where planned as well, the reasons for admission were mixed (i.e., surgical, investigation and medical). Patients with a cardiac condition may feel more acutely at risk of rapid deterioration, and perhaps this finding reflects the perceptions of the anticipated possible outcomes between conditions. If true, this highlights the importance of pre-admission education about the possible outcomes and actions to be taken post-discharge [28, 29]. Research on technology use in healthcare has predominantly focused on the experiences of patients with particular conditions and on patients that have used technology [24]. In this study, we sought to understand the preferences of both those who do and who do not currently use technology in daily life and health management. Investigation of experiences across different conditions warrants further investigation in order to inform appropriate health service responses for patients with a variety of conditions.

Income may affect preferences for communicating about symptoms that are not of concern post-hospital discharge. Patients that reported earning over 100,000 AUD per household per year were more likely than respondents with lower incomes to prefer to use telephone or technology-enabled forms of communication. Earning over 100,000 AUD per household per year was also associated with having higher educational qualifications in this study. Higher-income and education have been previously associated with greater technology use to manage health, such as patient portals [30] and smartphone applications [31]. Some have suggested that there is an emerging ‘digital divide’ where patients that lack access to computers and smartphones for a variety of reasons could miss out on health innovations that use digital technology [32]. However, a recent review (albeit of few studies) reported no associations with patient characteristics (including income) and digital health tool use [33].

Age was significantly associated with different preferences in communication about symptoms. Older people were less likely to prefer using technology over attending in-person when they had symptoms not of concern. It has been reported previously that younger people are more likely to be users of mobile health applications [31], and older persons are less likely to access and use the internet. However, older people were more likely to prefer making a telephone call over attending in-person when they had a concerning symptom. This finding may be because older people have had more experience with illnesses and have had more time to develop an understanding of their conditions and health services [34–36]. Alternatively, older persons may seek to negotiate a delay in admission to the hospital [37]. One review of health-related decision-making in older adults found that limited research in this area exists, however, delays in treatment-seeking by older persons were noted [38]. The desire to remain independent, the influence of others, availability and perceptions of health services available, and having access to information may also affect decisions to seek treatment [39].

Several limitations should be considered in the interpretation of the results of this study. These results represent a small proportion of patients presenting for a planned admission at a metropolitan hospital that predominantly performs surgical or investigational procedures. Therefore, the preferences for communication may not be generalisable to other patients with either different characteristics, those receiving different types of interventions, or those receiving care at different facilities. There was a low completion rate compared to the number of patients admitted during the time of the survey. As participation in the study was voluntary, the results of this study may be biased towards patients that were more interested in the topic, those with more time in the admission area, and those less distracted or concerned by the admission. Data about the characteristics of non-respondents was not available for comparison. Therefore, there may be alternative views of the target sample population that are not represented in the results of this study. As understanding patient preferences for communicating with health service providers through digital technologies is an emerging topic, a review to standardise terms is encouraged to enable future studies to be compared. Differentiating types of communication (e.g. email, website) from the devise used (e.g. mobile phone, laptop) would also allow for a greater understanding of preferencing among those who prefer using technology to communicate with the healthcare team. Future research should measure actual behaviours and reasons for choice in communication with hospitals post-hospital discharge if alternative technologies are made available. It should also consider understanding a broader range of reasons patients may have for contacting health service providers and patient characteristics that may be amenable to facilitating the adoption of technology in healthcare. Differences in technology availability and utilisation across different hospital facilities should also be investigated.

### Implications for practice

The role of the patient partnering with health services is an evolving concept that is increasingly recognised as integral to the delivery of patient-centred and quality healthcare [24]. The use of technology in health will continue to expand, but this study highlights the need for considering the needs and preferences of patients for communicating about their health needs. As health services continue to develop, the move away from a paternalistic system towards self-advocacy, empowerment, and quality is likely to see patients demand increased choice and control about how and when they communicate with healthcare providers [40]. Our study here highlights that acceptance of technology for communication about health is not pervasive, and that service development should also be informed by the needs and preferences of the patients they serve. In addition, these findings perhaps signal that most patients may prefer not to return to hospital for routine follow-up care if they have little or no concerns about their recovery. Health services with protocols requiring in-person follow-up care when recovery is progressing as planned should re-evaluate this practice.

## Conclusion

This is the first study to evaluate for patient preferences for using ICTs to facilitate communication between patients and health service providers following their discharge from hospital. We found patients presenting for a planned admission to hospital use a variety of technologies to manage their daily activities and health. Patients also had preferences that varied depending on their concerns for communicating with the healthcare team post-hospital discharge. It demonstrates that some, but not all, patients may prefer to use technology to traditional methods for communicating with the healthcare team post-hospital discharge. The majority of patients currently prefer more traditional methods for communication about symptoms post-hospital discharge (i.e., in-person or by telephone). Preferencing not to visit the hospital in-person is preferred when symptoms experienced are not of concern to a patient. These findings are important in the context of increased pressure to use technology to deliver healthcare efficiencies. These findings reinforce that health services should offer patients multiple options for communicating about their recovery to ensure individual needs are met appropriately. To fully realise the potential for greater service delivery efficiency and enhanced patient satisfaction with the healthcare experience that ICT may provide, health services looking to introduce new technologies to assist people with their symptom management should collaborate with patients to ensure such investments are warranted and adopted.

## Supplementary Information

**Additional file 1.**

## Data Availability

The datasets used and analysed during the current study are available from the corresponding author on reasonable request.

## Abbreviations

ICT: Information and communication technology
ePROMs: Electronic patient-reported outcome measures
EMR: Electronic medical record
RRR: Relative Risk Ratio
CI: Confidence Interval
