# Supplementary material for: Patient preferences for using technology in communication about symptoms post hospital discharge
Source: BMC Health Serv Res. 2021 Feb 15;21:141. doi: 10.1186/s12913-021-06119-7 (PMC7882859; doi:10.1186/s12913-021-06119-7)
Supplement: Supplementary file 1 — Additional file 1. [file 12913_2021_6119_MOESM1_ESM.docx]

Additional File 1

**Use of Technology Survey**

In terms of your **general day to day activities**, please select one of the three options that best applies to you regarding your use of the following technologies:

|  | I currently use |  | I don’t use but would be interested in using |  | I don’t use and don’t have any interest in using |
| --- | --- | --- | --- | --- | --- |
| Laptop or desktop computer | □ |  | □ |  | □ |
| Tablet (e.g. iPad) | □ |  | □ |  | □ |
| Internet / website | □ |  | □ |  | □ |
| Email | □ |  | □ |  | □ |
| Mobile phone | □ |  | □ |  | □ |
| Home phone | □ |  | □ |  | □ |
| Text messages | □ |  | □ |  | □ |
| Mobile phone or tablet app | □ |  | □ |  | □ |
| Online social network sites (e.g. Facebook) | □ |  | □ |  | □ |
| Online discussion group or forum | □ |  | □ |  | □ |
| Tele/video conferencing (e.g. Skype) | □ |  | □ |  | □ |

In terms of **managing your health**, please select one of the three options below that best applies to you regarding your use of the following technologies:

|  | I currently use |  | I don’t use but would be interested in using |  | I don’t use and don’t have any interest in using |
| --- | --- | --- | --- | --- | --- |
| Laptop or desktop computer | □ |  | □ |  | □ |
| Tablet (e.g. iPad) | □ |  | □ |  | □ |
| Internet / website | □ |  | □ |  | □ |
| Email | □ |  | □ |  | □ |
| Mobile phone | □ |  | □ |  | □ |
| Home phone | □ |  | □ |  | □ |
| Text messages | □ |  | □ |  | □ |
| Mobile phone or tablet app | □ |  | □ |  | □ |
| Online social network sites (e.g. Facebook) | □ |  | □ |  | □ |
| Online discussion group or forum | □ |  | □ |  | □ |
| Tele/video conferencing (e.g. Skype) | □ |  | □ |  | □ |

Please rank in order (from 1 to 9) your preference for communicating with your healthcare team following your visit to the hospital when you have symptoms that you **are not** concerned about:

|  | In-person (face-to-face) |
| --- | --- |
|  | Telephone |
|  | Tele/video conference (e.g. Skype) |
|  | Online via laptop or desktop computer |
|  | Online via tablet (e.g. iPad) or mobile device |
|  | Text message |
|  | Email |
|  | Online via social network (e.g. Facebook) |
|  | Online via discussion group or forum |

Please rank in order (from 1 to 9) your preference for communicating with your healthcare team following your visit to the hospital when you have symptoms that you **are** concerned about:

|  | In-person (face-to-face) |
| --- | --- |
|  | Telephone |
|  | Tele/video conference (e.g. Skype) |
|  | Online via laptop or desktop computer |
|  | Online via tablet (e.g. iPad) or mobile device |
|  | Text message |
|  | Email |
|  | Online via social network (e.g. Facebook) |
|  | Online via discussion group or forum |
